# Combined effects of 5-Fluorouracil, Folinic acid and Oxaliplatin on the expression of carcinoembryonic antigen in human colon cancer cells: pharmacological basis to develop an active antitumor immunochemotherapy

**DOI:** 10.1186/1756-9966-27-5

**Published:** 2008-05-19

**Authors:** Salvatore P Prete, Mario Turriziani, Maria C Massara, Alessia De Rossi, Pierpaolo Correale, Liana De Vecchis, Francesco Torino, Laura Bonmassar, Angelo Aquino

**Affiliations:** 1 Department of Neuroscience, Section of Pharmacology and Medical Oncology, University of Rome, "Tor Vergata", Via Montpellier, 1. 00133 Rome, Italy; 2 Department of Internal Medicine, University of Rome "Tor Vergata", Rome, Italy; 3 Oncopharmacology Center, School of Medicine, University of Siena, Italy; 4 Division of Medical Oncology, San Filippo Neri Hospital, Rome, Italy; 5 Istituto Dermopatico dell'Immacolata (IDI, IRCCS), Rome, Italy

## Abstract

**Background:**

Five-fluorouracil (FU), mainly associated with leucovorin (L), plays an essential role in chemotherapy of colorectal carcinoma. Moreover, FU ± L has been found to increase the expression of tumor-associated carcinoembryonic antigen (CEA), that may be an important target in therapeutic protocols of active specific immunotherapy. FU + L (FUL) are frequently combined with oxaliplatin (OXA) in advanced colon cancer patients. Thus, we investigated whether FUL in combination with OXA according to 2 different schedules may influence CEA expression in human colon cancer cells in vitro.

**Methods:**

CEA protein expression was evaluated by cytofluorimetric and western blot analysis. Relative quantification of CEA mRNA was assessed by real time RT-PCR analysis.

**Results:**

Levels of CEA protein and transcript were found to be higher in FUL-treated cells than in controls. However, when target cells were exposed to OXA before but not after FUL treatment, the up-regulation of CEA was partially inhibited.

**Conclusion:**

These results suggest that target cells must be exposed to OXA after but not before treatment with the fluoropyrimidine in order to exploit drug-induced up-regulation of CEA. This finding appears to provide useful information to design chemo-immunotherapy protocols based on FUL + OXA, combined with host's immunity against CEA directed cancer vaccines.

## Background

Colorectal cancer is one of the most commonly occurring malignancies in the world, and the prognosis for patients with advanced colorectal cancer with distant metastasis may be very poor. Among the antitumor agents, 5-fluorouracil (FU) is considered the reference drug for the treatment of advanced colon cancer. It is considered equally active in the treatment of a number of other common malignancies such breast, gastric and head and neck cancer [1].

In the last 5 years, FU was more frequently used in combination with other agents endowed with different mechanism of action such as oxaliplatin (OXA) or irinotecan [2-4] with which the antimetabolite has shown significant synergistic interaction. FU has also been associated with biotherapy, e.g. with monoclonal antibodies directed against vascular endothelial growth factor, such as Bevacizumab [5], or to the epidermal growth factor receptor, such as Cetuximab [6]. In advanced colorectal cancer, combination therapy appeared to provide better results in terms of response rate and survival, respect to treatment with FU alone [1-8]. These clinical trials, however, did not show a noticeable cure rate or long term survival, and there is a need for the development of new treatment modalities, possibly involving specific immunotherapy. Therefore, vaccine approaches has been proposed in order to obtain eradication of target malignant cells. Actually, a large variety of tumor-associated antigens (TAAs) has been identified, allowing the development of several tumor vaccines that are currently under investigation [9].

Carcinoembryonic antigen (CEA) is a 180,000 – 200,000 M_r _glycoprotein, widely used as a human tumor marker. CEA is present in various types of neoplasm, including gastrointestinal, breast and lung carcinoma [10]. Since this protein is scarcely expressed in normal tissues, whereas moderate/high CEA levels are often present in malignant cells, this tumor marker is considered a self TAA and a target for immunotherapy [11,12]. A number of clinical studies established that different strategies of immunization, including recombinant viruses, DNA or antiidiotypic antibodies can generate a CEA-specific cytotoxic T lymphocyte (CTL) response with anti-tumor activity in cancer patients and in mice [13-15].

An in vitro human CTL-mediated response to CEA has been demonstrated using CEA-derived immunogenic peptides (CEA-DIP) endowed with HLA-A(*)02.01 binding anchor motifs. One of these peptides (i.e. CAP-1) was found to elicit CTL responses in HLA-compatible human peripheral mononuclear cells (PBMC), obtained from normal donors or from patients with colorectal carcinoma [16].

Clinical trials are currently evaluating the possibility of using CEA as target antigen in protocols of active specific immunotherapy of patients with colorectal carcinoma. So far, the preliminary results of these investigations did not show the expected satisfactory results. It is possible that a sufficiently large fraction of the entire tumor cell population would not express adequate amounts of CEA-DIP to be recognized by specific CTLs. Therefore, agents capable of increasing the expression of CEA might represent suitable tools to improve the efficacy of therapeutic approaches, based on this tumor marker.

Our laboratory and other research groups have already demonstrated that the level of CEA can be increased by a number of therapeutic agents, including antitumor compounds (drug-induced antigen remodeling, DIAR) [17]. In addition, FU treatment was found to enhance cancer cell susceptibility to CEA specific CTL lines, generated from human HLA-A (*)02.01-positive PBMC stimulated in vitro with autologus dentritic cells pulsed with CEA-DIP [18].

Recently, immune adjuvant regimen with interleukin-2 and granulocytes macrophage colony stimulating factor has been tested in colon cancer patients in combination with a FU-based chemotherapy regimen, termed "GOLF" (i.e. gemcitabine; OXA; L and FU). The results of this study have been encouraging, since this immuno-chemotherapy protocol showed a powerful anti-tumor activity in metastatic colon cancer patients along with detectable increase of circulating tumor-specific CTL precursors [19].

These results prompted us to investigate the influence of OXA and FU, alone or in combination on CEA expression in "in vitro" model of human colon cancer cells.

## Methods

### Cell cultures

Human colon cancer cell line HT-29 was obtained from American Type Culture Collection (Rockville, MD). Tumor cells were routinely grown in Dulbecco's Modified Eagle Medium (DMEM), supplemented with 2 mM L-glutamine, 100 U/ml penicillin, 100 μg/ml streptomycin, and 10%, heat-inactivated, FCS (hyclone), hereafter called "Complete Medium" (CM). At each line passage, cells were removed using trypsin-EDTA solution [0.05% trypsin and 0.02% ethylenediaminetetra-acetic acid (EDTA) in phosphate buffered saline (PBS) without calcium and magnesium].

### Tumor cell treatment

Sub-confluent cells were removed by treatment with trypsin, counted and 5 × 10^6 ^cells were seeded in T75 flasks in the final volume of 15 ml of CM (day 0) and incubated at 37°C in an atmosphere of 5% CO_2 _and 90% humidity for 24 h. Thereafter, tumor cells were exposed to FU (10^-5^M) + L (10^-4 ^M) (i.e. FUL), or to OXA (10^-4 ^M) alone, or to FUL + OXA. This was done since preliminary studies on the interaction between FU and OXA, showed that the platinum compound was able to reduce the effect of the fluoropyrimidine on CEA expression when tumor cells were exposed to the 2 agents at the same time. In this study, all treated groups were exposed to FUL for 2 days (i.e. day 1 and 2), alone or combined with OXA, according to 2 different schedules: (a) cell cultures were incubated with OXA for 4 h in the morning of day 1, washed and treated with FUL (i.e. OXA on day 1), or (b) OXA was added to cell cultures on the morning of day 2, left for 4 h and subjected to multiple washing. Thereafter, cultured cells were re-exposed to FUL at the same concentration until day 3. On day 3 the cells were detached by using trypsin/EDTA, washed three times with PBS, counted and tested for CEA.

### PolyA (+) RNA isolation and Real-Time RT-PCR

PolyA(+)RNA was isolated using a Dynabeads direct micro-kit (Dynal, Oslo, Norway). mRNA was eluted in 10 mM Tris-HCl (10 μl) after incubation of the Dynabeads-mRNA complex at 92°C for 2 min. cDNA was synthesized using 10 μl of eluted mRNA and TaqMan RT kit (Applied Biosystems, Foster City, CA), according to the manufacturer's instructions. Real-time RT-PCR was performed in triplicate using the ABI PRISM 7700 Sequence Detection System (Perkin-Elmer, Wellesley, MA), according to the manufacturer's instructions. The two primers (forward, 5'-AGGACCACAGTCACGACGATC-3'; reverse, 5'-CTGGTGATGAAGGGTTTGGG-3') and the TaqMan probe (5'-CAGTCTATGCAGAGCCA-3') were specifically designed for the CEA gene using Primer Express v1.5a (Applied Biosystems). cDNA were amplified using PCR Master Mix according to the following PCR conditions: 50°C for 2 min, 95°C for 10 min, followed by 40 cycles at 95°C for 15 s and 60°C for 1 min. To correct for the variation of CEA mRNA value, real time RT-PCR analysis of glyceraldehyde-3-phosphate dehydrogenase (GAPDH) was carried out using the TaqMan GAPDH control reagents kit (Applied Biosystems).

### Relative Quantification Method

The relative quantification of CEA was performed using the comparative threshold cycle (C_T_) method (as described by "Applied Biosystems") that uses an arithmetic formula (2^-ΔΔCT^), which requires equal PCR_s _efficiency. ΔC_T _is the difference in the C_T _values between the target carcinoembryonic antigen (average C_T _used, as each sample was analyzed in triplicate) and the endogenous control GAPDH. ΔΔC_T _is the difference between ΔC_T _of a sample and the ΔC_T _of a calibrator sample. The mRNA obtained from HT-29 cells was chosen as calibrator sample.

### Cell extraction and electrophoresis

Cells were washed with PBS. The cell pellet was suspended in 3 volumes of extraction buffer [(50 mM Tris-HCl, pH 7.5-1 mM Phenylmethylsulfonyl Fluoride – 2 mM ethylene glycol-bis(beta-amino-ethyl ether)-N, N, N', N'-tetra-acetic acid – 400 mg/ml soybean trypsin inhibitor – 10 mM dithiothreitol – 5 μg/ml of aprotinin – 200 μg/ml of leupeptin – 1% Triton X-100], kept on ice for 10 min, sonicated for 5 sec and centrifuged for 5 min at 15,000 × g at 4°C in an Eppendorf microcentrifuge. Protein concentrations were determined with the Bio Rad protein reagent with bovine serum albumin as the standard. Proteins were heated in a boiling water bath for 2 min, and separated in 10% sodium dodecyl sulfate (SDS) (w/v) polyacrylamide gels using a mini-protean electrophoresis apparatus (BioRad, Hercules, CA, USA). All reagents were obtained from Sigma.

### Immunoblotting

The BioRad mini Trans-blot apparatus for electrophoresis was used for electrotransfer of proteins to nitrocellulose filters. The transfer was carried out at 25 V overnight. After the transfer, membranes were incubated with 3% of non-fat dry milk (Bio-Rad) in Tris buffered saline (TBS) (20 mM Tris-HCl pH 7.5, 0.9% NaCl) with gentle agitation, for 30 min. The membranes were then incubated at room temperature for 30 min, with COL-1 mAb (kindly provided by J.W. Greiner, National Cancer Institutes, National Institutes of Health, Bethesda USA) diluted (14 μg/ml) in TBS containing 0.05% Tween 20 (TBST), washed twice with TBST and incubated with alkaline phosphatase-coupled secondary antibody diluted 1:7,500 in TBST for 30 min. The bands were visualized using the Protoblot (Promega Biotec, Madison, WI, USA) color development system, as described by the manufacturer.

### Flow cytometry

Cancer cells were harvested with trypsin-EDTA solution, washed twice with PBS containing 0.02% sodium azide (Sigma) and distributed into 3-ml tubes (10^6 ^cell/tube). The cells were incubated with an excess of the primary anti-CEA monoclonal antibody (mAb) COL-1 in ice bath for 30 min, followed by two washes in PBS containing sodium azide. The cells were again incubated in ice bath for 30 min, washed twice with cold PBS and analyzed using a FACScan instrument (Becton-Dickinson, Rutherford, NJ) equipped with a blue laser with an excitation of level of 15 nW at 488 nm and the Lysis II program. Percentage of fluorescence of 10.000 cells was recorded and the background control of individual samples was subtracted. Data collections were gated utilizing forward light scatter and side light scatter to exclude the minimal of cell debris and aggregates. Data analysis was performed by using "Consort 32" software on a Hewlett Packard computer.

## Results

### Influence of OXA treatment before or after exposure to FUL on the growth and CEA expression of HT-29 cells

**Figure 1:**
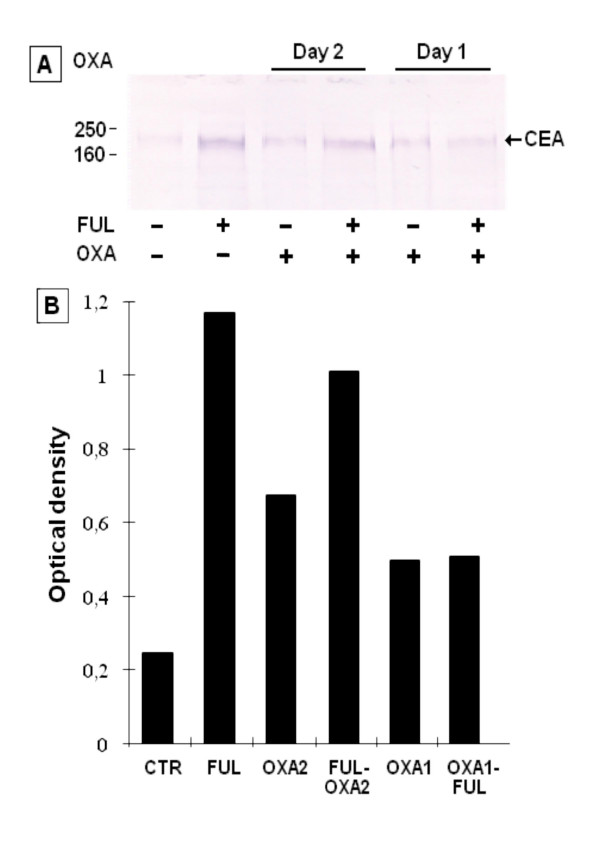
**Western blot (A) and densitometric analysis (B) of CEA expression in HT-29 cells.** Following treatment with oxaliplatin (OXA) before or after exposure to 5-fluorouracil + leucovorin (FUL), cells were washed in PBS and the pellet was suspended in extraction buffer. Cell lysates were subjected to SDS-PAGE and immunoblotting was performed with MAb COL-1 for CEA detection. Molecular weights in kDa are placed on the left. See "material and methods" for experimental details. Immunoblot was scanned by densitometer and the optical density was expressed as arbitrary units. OXA2, cells were treated with OXA on day 2; FUL-OXA2, FUL followed by OXA2 treatment; OXA1, cells were treated with OXA on day 1; OXA1-FUL, OXA1 followed by FUL treatment.

Preliminary experiments suggested possible negative interactions between FU and OXA on CEA expression, when tumor cells were exposed to the 2 agents together. Therefore, we decided to investigate the effect of FUL, alone or combined with OXA on HT-29 colon cancer cells, using different schedules of the two drugs, i.e. OXA given before FUL (OXA day 1) or OXA given after 1-day exposure to FUL (OXA day 2). This time schedule was adopted in order to explore whether 1 day separation between FUL and OXA treatment could be adequate to prevent negative drug interaction on tumor marker expression. On day 3, the number of viable cells (i.e., cells excluding trypan blue) was counted and subjected to western blot (Figure 1) and FACS analysis.

**Figure 2:**
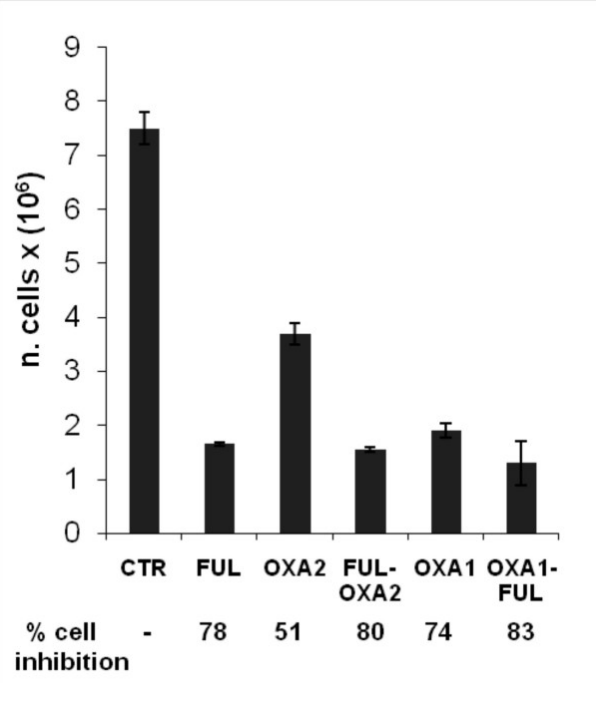
**Effects of oxaliplatin (OXA) and 5-fluorouracil+leucovorin (FUL) on the HT-29 cells.** Cells were treated with OXA on day 1 before exposure to FUL or on day 2 after 24 h exposure to FUL. Treatment conditions are described in "Material and Methods". On day 3, cells were counted by trypan blue exclusion and CEA expression was measured by FACS analysis by using COL-1 mAb. Columns, total number of cells. Bars, SE of triplicate determinations. No significant difference was observed between the effects of FUL-OXA2 versus OXA1-FUL (p > 0.05). The percentage of the CEA-positive cells was as followed: 14.9 (untreated control); 30.1(FUL); 19.9 (OXA2); 26.2 (FUL-OXA2); 19.1 (OXA1); 21.4 (OXA1-FUL). OXA2, cells were treated with OXA on day 2; FUL-OXA2, FUL followed by OXA2 treatment; OXA1, cells were treated with OXA on day 1; OXA1-FUL, OXA1 followed by FUL treatment.

Densitometric analysis of immunoblot (Figure 1B) indicated that CEA levels were 4.7 fold higher in HT-29 cells treated with FUL, in comparison to untreated controls. Treatment with OXA alone, on day 1 or day 2, induced a 2.7 or 2.0-fold increase, respectively, of CEA expression compared with untreated cells. However, when the cells were exposed to OXA on day 1, before FU treatment, the up-regulation of CEA was inhibited (Figure 1A, B). On the contrary, when HT-29 cells were treated with OXA on day 2 (i.e. 1 day after exposure to FUL), the platinum compound did not reduce sensibly the FUL-mediated up-regulation of CEA expression, which remained on the value of approximately 4-fold increase. Western blot analysis evaluated the total CEA content. Therefore, the expression of CEA on the cell membrane was determined by using FACS analysis. The results of a typical experiment illustrated in the legend of figure 2, indicate that the up-regulation of membrane-associated CEA was partially inhibited when the cells were exposed to OXA before, instead of after FUL treatment.

The results, illustrated in figure 2 showed that treatment with FUL is followed by 78% reduction of the total number of cells. The sequential treatment of OXA before FUL exhibited 83% of cell inhibition. No differences in the growth rate were detected by using the reverse sequence of drug combination (i.e. FUL before OXA).

### Effect of FUL and OXA alone or in combination on CEA transcripts

Parallel experiments were performed in order to test whether the increase of CEA protein resulting from drug treatment of HT-29 cells with FUL and OXA alone or in combination, could have also resulted in an increase of CEA mRNA. Detection of CEA transcripts was carried out by real time RT-PCR using the CEA specific probe and primers, as described in "Materials and Methods". The efficiency of amplification of CEA and GAPDH were found to be equal. This enabled relative quantification of CEA to be performed using the ΔΔC_T _comparative method illustrated in the section of "Material and Methods"

**Figure 3:**
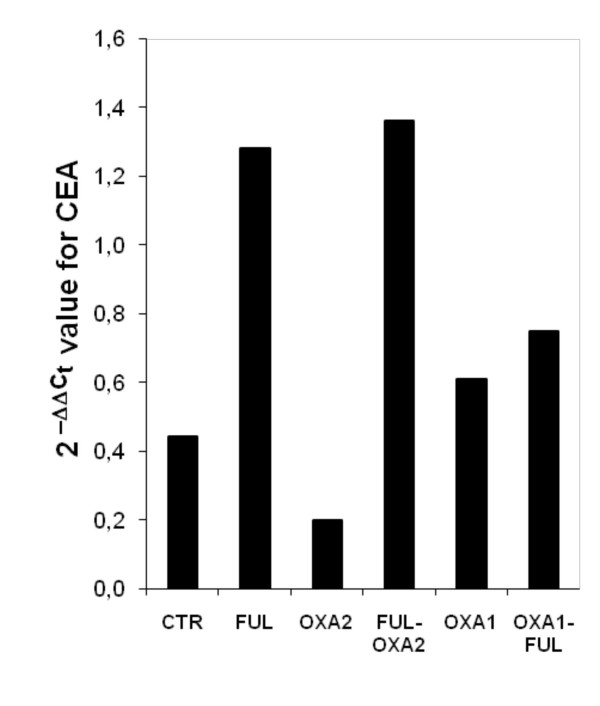
**Relative CEA mRNA values of cells treated with 5-Fluorouracil+leucovorin (FUL) and/or Oxaliplatin (OXA).** Details of cell treatment are illustrated in "Materials and Methods". The relative CEA mRNA values were measured by Real time RT-PCR and calculated using the comparative C_T _method as described in "Materials and Methods". OXA2, cells were treated with OXA on day 2; FUL-OXA2, FUL followed by OXA2 treatment; OXA1, cells were treated with OXA on day 1; OXA1-FUL, OXA1 followed by FUL treatment.

The results described in Figure 3, showed that CEA transcripts were 2.9 times higher in HT-29 cells treated with FUL compared with those of untreated cells. Noteworthy, CEA mRNA was found in a 3.1-fold greater abundance in cells treated with FUL followed by OXA on day 2 in comparison to those in untreated cells. On the contrary it was revealed only a 1.7 fold increase in the level of CEA when the cells were exposed to OXA on day 1 before FUL treatment.

## Discussion

The results of the present study revealed a complex interaction between FUL and OXA, a triad of agents largely used in colorectal cancer, on CEA expression of human HT-29 colon cancer cells in vitro. From one side, our findings confirm previous observations on the increase of CEA expression by FUL, either at protein and transcript levels [20]. On the other side, the data illustrated in this report, showed a marginal increase of CEA expression in target cells treated with OXA alone, respect to that detectable in untreated controls. In contrast, exposure of cancer cells to OXA prior to FUL treatment, antagonized consistently FUL-induced up-regulation of the antigen. However, this negative interaction did not appear to take place when target cells were exposed to the platinum derivative 24 h after treatment with the fluoropyrimidine. Worth of note is the observation that the short-term cytotoxic effects of drug combination were essentially unaffected by the order of OXA – FUL sequence. Moreover previous investigations showed that FU and L treatment acted in synergy to inhibit the growth of tumor cells in vitro but did not alter CEA modulation induced by the antimetabolite [20]. All these observations support greatly the hypothesis that drug influence on CEA expression is not correlated with tumor suppressive effects of these agents.

No data are presently available on the molecular bases involved in the interaction between FU, L and OXA on the expression of CEA.

It reasonable to hypothesize that the increase of CEA mRNA observed after FUL treatment (see Figure 3) is responsible, at least in part, of the enhanced CEA protein levels as shown by the results of Figure 1. This is further supported by previous findings demonstrating that treatment with Actinomycin D, an inhibitor of RNA synthesis, blocked the enhancement of CEA transcript in FU-treated cells [18]. However, the actual biochemical mechanism underlying FU-mediated increase of CEA is entirely unknown. In any case, the hypothesis that selection of CEA positive FU-resistant cells could play a role, does not appear to be easily acceptable. In fact, FU-induced increase of CEA levels was obtained also in C22.20 cell line derived by sub-cloning a clone of HT-29 cells [21], thus indicating that CEA up-regulation is based on biochemical induction rather than selection.

The role possibly played by drug-induced up-regulation of CEA expression deserves particular attention. Actually, it has been demonstrated that immunogenic peptides, generated by endocellular processing of proteins predominantly located in malignant cells, can be presented in association with HLA molecules to responder host's lymphocytes. In the present model, CEA-DIP has been shown to elicit efficient cell mediated immunity, with generation of HLA-restricted CTL [13,16]. Moreover, animal studies demonstrated synergistic effects between antitumor chemotherapy and immune responses, even when host's responses are particularly weak [22]. Since drug-induced up-regulation of CEA is followed by consistent increase of CEA-DIP [18], this phenomenon could be exploited for chemo-immunotherapy of CEA-positive neoplastic diseases. Many CEA-based cancer vaccines approaches are currently being evaluated in clinical trials involving patients with malignant diseases of the digestive tract. The preliminary results have shown that it is possible to induce an effective antigen-specific cellular and humoral response [9,13,14,16]. However, so far, no correlation has been demonstrated between immunostimulation and clinical outcome. One of the possible explanations is that CEA is heterogeneously expressed in the tumor. Therefore, some of the neoplastic cells may escape recognition by vaccine-induced CEA-specific CTLs because of reduced expression of target antigen. In the light of this hypothesis, pharmacological intervention able to induce changes of the antigenic-immunogenic profile of tumor cells is of potential clinical interest. We and other authors previously reported that treatment with different compounds (e.g. with fluoropyrimidine, staurosporine, interleukin-6 and interferons) may induce CEA up-regulation [17,23]. A similar event has also been observed for the expression of class-I HLA molecules [20]. Moreover, CEA expression enhancing agents are able to increase the level of this antigen in various clones expressing different basal level of the marker, including clones expressing only marginal amounts of CEA protein [24].

In conclusion, the rationale to utilize the optimal treatment schedule of the FUL-OXA combination capable of inducing both growth inhibition and CEA up-regulation, appears to be of considerable value, since drug-induced overexpression of the antigen is expected to make tumor cells more susceptible to the cytolytic activity of specific effector lymphocytes.

## Competing interests

The authors declare that they have no competing interests.

## Authors' contributions

SPP carried out the cytofluorimetric studies. MT performed the experiments on tumor cells treated with drugs. MCM contributed to real time RT-PCR analysis. ADR carried out the immunoblotting analysis. PC has been involved in revising the manuscript. LDV participated in the designed of the studies. FT helped to draft the manuscript. LB has been involved in molecular biology studies. AA supervised experimental work and manuscript editing. All authors read and approved the final manuscript
